# Permissibility, Moral Emotions, and Perceived Moral Agency in Autonomous Driving Dilemmas: An Investigation of Pedestrian-Sacrifice and Driver-Sacrifice Scenarios in the Third-Person Perspective

**DOI:** 10.3390/bs15081038

**Published:** 2025-07-30

**Authors:** Chaowu Dong, Xuqun You, Ying Li

**Affiliations:** School of Psychology, Shaanxi Normal University, Xi’an 710062, China; dongchaowu@snnu.edu.cn (C.D.); youxuqun@snnu.edu.cn (X.Y.)

**Keywords:** automated vehicles, moral dilemma, moral decision-making, moral emotion, perceived moral agency

## Abstract

Automated vehicles controlled by artificial intelligence are becoming capable of making moral decisions independently. This study investigates the differences in participants’ perceptions of the moral decision-maker’s permissibility when viewing scenarios (pre-test) and after witnessing the outcomes of moral decisions (post-test). It also investigates how permissibility, ten typical moral emotions, and perceived moral agency fluctuate when AI and the human driver make deontological or utilitarian decisions in a pedestrian-sacrificing dilemma (Experiment 1, *N* = 254) and a driver-sacrificing dilemma (Experiment 2, *N* = 269) from a third-person perspective. Moreover, by conducting binary logistic regression, this study examined whether these factors could predict the non-decrease in permissibility ratings. In both experiments, participants preferred to delegate decisions to human drivers rather than to AI, and they generally preferred utilitarianism over deontology. The results of perceived moral emotions and moral agency provide evidence. Moreover, Experiment 2 elicited greater variations in permissibility, moral emotions, and perceived moral agency compared to Experiment 1. Moreover, deontology and gratitude could positively predict the non-decrease in permissibility ratings in Experiment 1, while contempt had a negative influence. In Experiment 2, the human driver and disgust were significant negative predictor factors, while perceived moral agency had a positive influence. These findings deepen the comprehension of the dynamic processes of autonomous driving’s moral decision-making and facilitate understanding of people’s attitudes toward moral machines and their underlying reasons, providing a reference for developing more sophisticated moral machines.

## 1. Introduction

In “*Paradise and Iron*,” the author envisioned “Sappho,” a vehicle without steering wheels, gears, or ignition, capable of fully autonomous driving (Breuer, 2020). As artificial intelligence (AI) rapidly develops, the convergence of the automotive industry and AI has become a trend in recent years. Automated vehicles (AVs) driven by AI will bring us more convenience and safety. AI empowers autonomous driving systems (ADSs) to access real-world information and then reason and decide what to do with the help of technologies (Chen et al., 2023), supporting AVs to assess and react to the surrounding environment instantly (Ma et al., 2020). Before widely deploying AVs, it is necessary to clarify the moral issues of AVs to ensure that the technology meets the needs of human society (Klenk, 2022). Thus, it is urgent to investigate what AI should do in moral dilemmas and how people perceive AI moral agents before they replace human drivers.

### 1.1. The Moral Decision-Making of AVs

Moral decision-making refers to various types of decisions made within the moral domain regarding moral issues or principles such as justice and harm, including choices of behavioral responses to moral dilemmas and judgments, or evaluations of others’ actions or moral character (Turiel, 1983). The process of moral decision-making involves evaluating possible actions and outcomes in response to a moral dilemma, ultimately determining which action aligns with social norms and values (Garrigan et al., 2018). It involves responding to environmental and social contextual information, evaluating affective emotions, and assessing the acceptability of actions and the characters involved. Deontology and utilitarianism are two predominant moral beliefs (Greene et al., 2001). Deontology emphasizes the importance of the process and insists on adhering to fundamental rights and obligations. It aims to avoid intentionally harming innocent people (Kant, 1785/1976). Utilitarianism emphasizes the outcomes of decisions, focusing on the greatest benefit (Bentham, 1789/1967). It allows intentional harm for the greater good of the majority.

The development of artificial intelligence enables machines to gradually participate in moral decision-making, becoming moral proxies and even machine moral agents (Bonnefon et al., 2024). For example, AI can make critical medical decisions (Laakasuo et al., 2025) and make judicial judgments (Kim & Peng, 2025). However, previous research has found that people are averse to machines making moral decisions, and this aversion mainly stems from the perceived lack of an intact mind—that is, the ability to think and feel (Bigman & Gray, 2018). Even when machines make the right decision for the greater good, people still believe that it is more appropriate for humans to make decisions. Thus, the primary question is whether machines should become moral decision-makers. Moreover, to design a moral machine that can be widely accepted, it is necessary to consider people’s preferences regarding the machines’ moral beliefs to reduce their aversion to machines making moral decisions.

As a frontier field of automated applications, the issue of moral decision-making in autonomous driving scenarios has received extensive attention. Awad et al. (2018) revealed the public’s preference for utilitarianism, which indicated a preference for saving more lives, including humans and younger individuals, over animals in driving moral dilemmas. Faulhaber et al. (2019) confirmed the majority’s pursuit of minimizing collision casualties, indicating a preference for utilitarianism. Moreover, when AVs made utilitarian decisions like participants did, trust in the AVs significantly increased (Yokoi & Nakayachi, 2021b). However, this trust is insufficient to mask the negative perception of harm caused to innocent individuals by AVs. Compared with most people who preferred utilitarianism, those who preferred deontology trusted human drivers more, and even AVs shared the same beliefs (Yokoi & Nakayachi, 2021a). According to Yokoi and Nakayachi (2021c), this trust bias arises because participants believe that AVs lack empathy, which prevents them from feeling what potential victims feel, especially when it involves the sacrifice of drivers in the car. In self-sacrificing studies, participants consistently hold a self-protective preference, for they often play the role of drivers in the experiments. Therefore, participants preferred deontological AVs, which protect drivers’ lives in moral dilemmas and take manual control more frequently when they thought AVs might sacrifice themselves (Ju & Kim, 2024; Pérez-Moreno et al., 2025). Since AVs still belong to the driver, they perceive AVs as personal property. Thus, AVs should prioritize the safety of their owners over others (Kallioinen et al., 2019; Liu & Liu, 2021; Liu et al., 2025). Indeed, participants considered utilitarianism to be less moral, more shameful, and blameworthy in self-sacrificing dilemmas (Bruno et al., 2023). Existing research has documented people’s general attitudes toward AI-controlled AVs, but it lacks an investigation into dynamic changes in the whole procedure, leading us to pose the following questions:

Q1: Do people’s perceived permissibility of decision-makers change after they witness the outcomes?

Q2: If such changes exist, what factors contribute to these changes?

To address these questions, this study aims to examine differences in permissibility judgments before and after individuals witness the outcomes. Drawing on dual-process theory, it investigates differences in emotional responses and perceived moral agency, as well as which factors could prevent a decrease in permissibility.

### 1.2. Moral Emotions and Moral Decision-Making

Moral emotions affect the reference frames of moral decision-making and the stability of decision outcomes (Gangemi et al., 2025). Haidt (2003, p. 853) defined moral emotions as “emotions linked to the interest or welfare of society as a whole or at least of persons other than the judge or agent.” Moral emotions underpin the capacity for morality (Lockwood et al., 2025), primarily including other-praising emotions, other-suffering emotions, other-condemning emotions, and self-conscious emotions based on their object and nature (Greenbaum et al., 2020).

Firstly, the other-praising emotions are “positive feelings that occur when another person upholds moral standards”, which include gratitude and elevation (Greenbaum et al., 2020). Gratitude arises from receiving benevolent assistance from others, which catalyzes reciprocal behaviors and the consolidation of interpersonal bonds. High-quality expressions of gratitude increase the likelihood of helpers assisting again in the future (Iwai & de França Carvalho, 2022). People who feel gratitude are more likely to pursue equity and altruism (Oh et al., 2023). Elevation arises when individuals observe others’ moral excellence, making observers feel warm and uplifted (Haidt, 2003). Notably, the observers are not the recipients of the kindness, so elevation would motivate them more to be a moral exemplar (Vianello et al., 2010). This effect is more substantial when the helper is perceived as an in-group member (Telesca et al., 2024). Moreover, experiencing elevation makes people pay more attention to moral behavior, which promotes adherence to deontology and a predisposition towards “dignified” rather than utilitarian decisions (Strohminger et al., 2011). Pride arises from the self-attribution of success (Weiner, 1985). When people believe that their behavior aligns with social standards or reflects their worth, they will feel pride (Mascolo & Fischer, 1995). Genuine pride is a positive emotion with positive self-evaluation (Kornilaki & Chlouverakis, 2004). It indicates the degree to which positive behavioral outcomes are associated with the individual and the group to which they belong. The stronger the association, the greater the pride people will experience (Tracy & Robins, 2006). As the cost of moral behavior increases, people experience more pride, and the probability of engaging in ethical behavior again also rises (Etxebarria et al., 2019). Bagozzi et al. (2022) stated that when people witness others engaging in friendly interactions with machines, such as treating robots kindly and praising and thanking them, they also feel pride.

Secondly, the other-suffering emotions are the feelings that occur “when another person is the victim of a moral standard violation” (Greenbaum et al., 2020). They typically include sympathy, compassion, and empathy, and these emotions tend to have similar definitions and are often used interchangeably (Goetz et al., 2010). Here, we adopt the term “sympathy” to denote the process from cognitions through emotions to behaviors, as Greenbaum et al. (2020) did, which also aligns with the Chinese linguistic context. Sympathy is an emotional response to the suffering of others, accompanied by a desire to help (Greenbaum et al., 2020). As an inter-subjective emotion, sympathy is vital to stabilizing social cooperation (Ye et al., 2010). It minimizes conflicts by reducing antagonism and fostering amicable behaviors, thereby enhancing collaboration (Dasborough et al., 2020). Generally, sympathy helps facilitate people’s interactions with others and informs our decision-making capabilities (Hardy, 2019). In moral decision-making, sympathy is positively related to the importance of dilemmas, which can predict both care (positively) and justice (negatively) orientations (Skoe et al., 2002).

Thirdly, the other-condemning emotions are negative feelings towards those who violate moral standards. Shweder et al. (1997) proposed that emotional responses are paired with specific social norm violations in the “CAD (contempt, anger, disgust)” triad hypothesis. Contempt arises from others violating moral standards without opportunities for reconciliation (Greenbaum et al., 2020). It is a disdain or disapproval towards moral norm-breakers, focusing on judgments involving descriptions of bodily harm (Landmann & Hess, 2017). As a judgment of the principal quality, particularly of incompetent or unintelligent decision-makers (Hutcherson & Gross, 2011), contempt could also predict the severity of punishment and help establish the judges’ reputations (Ginther et al., 2022). Anger arises from the unreasonable violation of justice (Haidt, 2003), igniting the desire to correct the injustice. Its intensity depends on the relevance between moral events and moral agents (Hutcherson & Gross, 2011). The stronger the anger, the less moral reasoning occurs. People who experience anger are more likely to adopt a more self-centered perspective, prioritizing personal interests over societal norms (Barger & Pitt Derryberry, 2013). Bruno et al. (2023) found that participants experienced a greater intensity of anger when the moral decision involved self-sacrifice, suggesting that when the moral agent chose to sacrifice themselves for the greater good, it elicited a sense of injustice or outrage, thereby intensifying stronger responses. Disgust arises from specific violations of moral sanctity and purity (Shweder et al., 1997) towards individuals and groups. It reflects the expectation of behaving as a respectable moral example. Since the standards of personal feelings of disgust are different (Ong et al., 2014), Białek et al. (2021) suggested using the subjective feelings of disgust as the measurement of the effect of disgust on moral judgment. As Hutcherson and Gross (2011) suggested, moral disgust should generally be an adaptive response to moral violations. For example, disgust has been found to highly correlate with deontological decisions in moral dilemmas (Szekely & Miu, 2015). Individuals with high disgust sensitivity are more susceptible to deontological guilt inductions, leading to a decrease in moral self-image and an increase in selfish and immoral behavior (Parisi et al., 2021).

Lastly, self-conscious emotions are “negative feelings towards the self because one has violated moral standards”, including shame, guilt, and fear. Shame arises from negative self-evaluations of one’s moral character, which are associated with self-perceived moral failures and cause feelings of unworthiness and social isolation. Experiencing shame can motivate individuals to engage in prosocial behaviors, thereby restoring their self-esteem and reconnecting with others (Greenbaum et al., 2020). Notably, shame differs across diverse populations, contexts, and cultures. For example, shame may also lead to defensive responses, such as self-blame or withdrawal from social interactions. Guilt arises from people’s moral failures, characterized by remorse and a desire to make amends (Ohbuchi, 1988). It motivates individuals to take responsibility for their moral behaviors and rectify the harm that they cause (Higgs et al., 2020; Haidt, 2003). Similar to shame, guilt is also highly dependent on social context. It has been linked to increased prosocial behavior as individuals seek to compensate for the harm that they have caused (Hutcherson & Gross, 2011). Moreover, both moral shame and guilt could motivate prosocial behavior. Most violent offenders lack a sense of shame and guilt, reporting violence as a morally acceptable and natural choice of action (Trivedi-Bateman, 2021). Fear arises from the potential for harm—encompassing physical, emotional, and psychological threats, as well as natural or imagined harm (Gray, 1987)—and is essential to survival (Niedenthal & Brauer, 2012). In moral decision-making, fear reflects individuals’ assessment of the intentionality of moral decisions (Harbin, 2020). It triggers biochemical and affective responses, facilitating moral reasoning and, ultimately, influencing the choice. Fear has been shown to increase the likelihood of utilitarian moral judgments in personal moral dilemmas (Tao et al., 2023). Additionally, fear also reflects individuals’ assessment of the intentionality of moral decisions (Harbin, 2020), especially during those “harm to save” moral dilemma scenarios, where participants experienced the most fear and sadness (Szekely & Miu, 2015).

In moral decision-making, conflicts between an individual’s or group’s beliefs and behaviors can lead to moral conflicts (Festinger, 1957), with moral emotions being a primary manifestation of such conflicts (Dahò, 2025). Haidt (2001) introduced the social intuitionist model, positing that moral judgments are predominantly based on intuition and immediate emotional responses rather than in-depth logical or rational analysis. Greene (2007) advanced the dual-process theory, suggesting that people continuously balance reasoning and emotions during moral decision-making. Since the ratio of reasoning and emotions varies across different scenarios, Moll et al. (2005) proposed the event–feature–emotion complex model, suggesting that reasoning and emotions are integrated throughout moral decision-making rather than competing (Helion & Ochsner, 2018; Moll & de Oliveira-Souza, 2009). With the advancement of technology, AI-controlled moral machines will soon be capable of perceiving, experiencing, and expressing emotions. Therefore, it is necessary to explore how people’s feelings towards moral machines compare with those of humans who hold different moral beliefs, which would aid in the development and refinement of moral machines.

### 1.3. Moral Agency and Moral Decision-Making

Moral agency refers to the capacity to comprehend moral norms, act in accordance with them, and assume responsibility for moral behaviors (Himma, 2009; Zafar, 2024). As moral agents, people engage in moral activities according to their moral agency in particular environments, especially in complex moral decision-making (Gonzalez Fabre et al., 2021). Therefore, people program machines to perform moral activities by imitating humans, using environmental information as a guide. Since the reasons for humans’ moral actions are complex and ambiguous, the human-made generative AI language models inevitably make mistakes and create logical relationships to obfuscate errors (Vallor & Vierkant, 2024). Thus, AI systems and machines should possess human-level moral agency before they replace humans in making moral decisions.

Moor (2006) distinguished ethical machines into ethical impact agents (“machine ethics, computers do our bidding as surrogate agents and impact ethical issues such as privacy, property, and power”), implicit moral agents (“the machine acts ethically because its internal functions implicitly promote ethical behavior-or at least avoid unethical behavior”), explicit moral agents (the machine “would be able to make plausible ethical judgments and justify them”), and full ethical agents (“a full ethical agent can make explicit ethical judgments and generally is competent to justify them reasonably”). From a technical perspective, the development of AI equips machines with the capacity for deep thinking, and intelligent systems are no longer merely simple agents with ethical implications. From a human perspective, full ethical agents are exclusively adults. Even the most advanced AI systems are rejected as full ethical agents due to their lack of consciousness, intention, and free will. In practical terms, AI can be either an implicit or explicit moral machine (Bonnefon et al., 2024). As an implicit moral machine, AI follows the rules coded in its program. For example, if an AI driver were coded to prioritize the safety of the driver, it would simply follow the rules and choose to sacrifice pedestrians when facing a moral dilemma. As an explicit moral machine, AI should be able to independently solve moral dilemmas. For example, when an AI driver needs to choose between the driver in the car and multiple pedestrians, it is more likely to prioritize the safety of the pedestrians over the driver. In the process of AI’s development from an implicit moral agent to an explicit moral agent, the actions of AI have moral repercussions, regardless of whether moral values are explicitly encoded. Since AI can learn moral norms through plausible engineering methods, it may be sufficient to cultivate morally responsible machine agency (Gogoshin, 2021).

The development of moral agency would bring more rational moral decision-making. For humans, the development of moral agency depends on personal experiences, cognitive abilities, and cultural context (Sugarman, 2005). Like humans, AI-controlled autonomous driving systems are becoming driving agents due to the advancement of devices and algorithms. They are gradually replacing human drivers in decision-making, particularly in emergencies requiring rapid responses (Bhattacharya et al., 2023). When AI makes moral decisions as a driver agent, it requires AI not only to hold human moral standards but also to possess moral agency to make moral judgments rationally and emotionally based on specific circumstances (Firt, 2024). From this perspective, only AI with high moral agency can effectively deal with various influencing factors, which would help protect human interests (Maninger & Shank, 2022) and solve the problem of responsibility allocation. AI needs to possess the same or even more autonomous reflection capacity than humans, evaluate and modify the content of its program, and make choices and act based on real-world situations (Swanepoel, 2021). When AI performs moral activities, its moral agency should match its intelligence and task complexity, as having a high degree of moral agency is one of the prerequisites for AI to deal with complex moral tasks effectively.

### 1.4. Current Research

**Figure 1:**
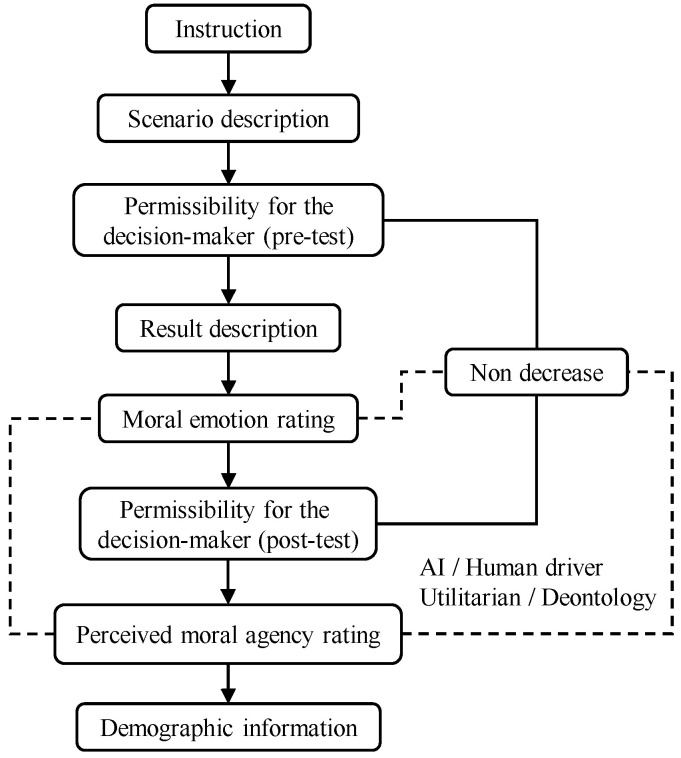
The procedure of Experiment 1 and Experiment 2.

The current research aims to achieve four objectives: (1) to investigate how the participants’ permissibility of the decision-maker fluctuates before and after witnessing the results through repeated measures (pre-test and post-test) under different decision-makers (AI, human driver) with different moral beliefs (utilitarianism, deontology); (2) to explore participants’ typical moral emotions under different decision-makers with different moral beliefs; (3) to investigate participants’ perceived moral agency when AI or a human driver makes utilitarian or deontological moral decisions; and (4) to explore which specific moral emotions and perceived moral energy could help maintain and improve the permissibility with a binary logistical regression (see Figure 1). Based on the driving moral dilemma scenarios used in existing studies, this study adopts two typical traffic moral dilemmas—the pedestrian-sacrificing dilemma and the driver-sacrificing dilemma—in the third-person perspective. We named the scenarios based on the outcomes of utilitarian choice. Study 1 uses the scenario involving the sacrifice of one pedestrian or two pedestrians, and Study 2 uses the scenario involving the sacrifice of one pedestrian or one driver.

## 2. Experiment 1

Experiment 1 investigated participants’ permissibility of different decision-makers and how different decision-makers with different moral beliefs affect permissibility, moral emotions, and perceived moral agency.

### 2.1. Materials and Methods

#### 2.1.1. Participants

A priori power analysis using G*Power 3.1 (Faul et al., 2007) with α = 0.05 indicated that 210 participants would provide 0.95 power, determining a medium-sized effect (f = 0.25; Cohen, 1992). Eight participants who failed the attention check task were excluded from the analysis. Two hundred and fifty-four participants (115 males, 139 females) completed the questionnaire for CNY 5. Their ages ranged from 18 to 45 (*M* = 24.85 years, *SD* = ±4.72 years). Two hundred and thirty-nine participants (94.09%) reported holding a driving license. One hundred and forty-one participants (55.51%) reported having experience with fully self-driving vehicles. The gender proportion differences across the four groups ranged from 5.1% to 16.4%. A chi-squared test revealed no significant difference (χ^2^(3) = 5.892, *p* = 0.117), indicating a balanced gender distribution among the groups.

#### 2.1.2. Experiment Design

Experiment 1 employed a between-subjects experimental design with a 2 (decision-maker: human driver “Mr. Y”, autonomous driving system “X”) × 2 (moral belief: utilitarianism, deontology) structure. The dependent variables included the participants’ ratings of the decision-maker’s permissibility (repeated measure), moral emotions, and perceived moral agency. Notably, the permissibility was rated at two time points: before and after the participants witnessed the outcomes.

#### 2.1.3. Procedure

**Figure 2:**
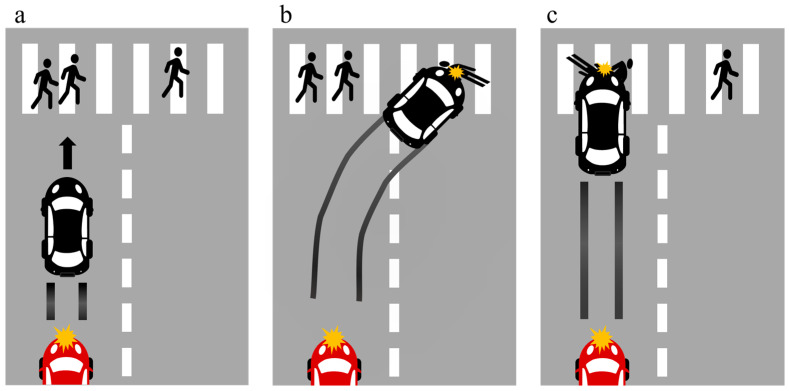
The scenario used in Experiment 1: (**a**) After being hit by the red car, the decision-maker needed to either make a sharp turn to the right or stay in the current lane. (**b**) The decision-maker makes a sharp turn to the right, running over the single pedestrian. (**c**) The decision-maker stays in the current lane, running over the two pedestrians.

An online survey platform, Credamo, was used to carry out the data collection procedure. To prevent display inconsistencies across devices, we only allowed the participants to complete the experiment on laptops or desktop computers. After reading the instructions and signing the informed consent form, the participants were asked to regulate their emotional states to a neutral level for at least 20 s to minimize emotional bias. After they considered their emotions to be calm, they were required to evaluate their emotional state with one item. Then, they were randomly assigned to one of four conditions: “Mr. Y” with utilitarianism, “Mr. Y” with deontology, “X” with utilitarianism, and “X” with deontology. Before the formal experiment, the participants needed to report their emotional states. Then, the scenario was presented: “Mr. Y is a seasoned driver, endowed with extensive driving experience and a safety record, having never been involved in a traffic accident throughout his driving career. The autonomous driving system ‘X’ can drive under all feasible conditions and matches the driving proficiency of the human driver ‘Mr. Y’. A black car is traversing the crossroad under normal circumstances. Suddenly, an out-of-control red car collides with it at high speed from behind, causing the black car to lose control. Both the driver, ‘Mr. Y’ and the autonomous driving system ‘X’ exert maximum effort to apply the brakes. However, they cannot reduce the speed to a safe level. At this moment, there are two pedestrians directly in front of the black car and one pedestrian on the right in front of the black car (Figure 2a). The only way to save the two pedestrians is to make a sharp turn to the right. Nevertheless, adopting this maneuver will result in the vehicle running over a single pedestrian (Figure 2b). If the black car keeps its current lane, it will inevitably run over the two pedestrians (Figure 2c)”. Following the reading task, the participants provided permissibility ratings for the decision-maker. Subsequently, four decision outcomes were presented to the participants (see Appendix A). The participants then reported the intensity of their moral emotions and revised the permissibility ratings for the decision-maker. The participants were required to recall the decision correctly to ensure proper comprehension of the scenario. Then, they needed to assess their perceived moral agency. After the participants had completed the demographic information, we informed them that the scenario was a rare event to reduce potential discomfort. Additionally, we described the scenarios from a third-person perspective to minimize the bias caused by emotional over-involvement.

#### 2.1.4. Measures

The items employed in Experiment 1 are detailed in Appendix B. All items were scored on a 5-point scale. Before the experiment, the participants rated their emotional states (1 = negative, 3 = neutral, 5 = positive (*M* = 3.96, *SD* = 0.98)). The results of a one-way ANOVA test showed no significant difference in the participants’ emotional states between the four groups before the experiment (*F*(3,250) = 0.86, *p* = 0.464, η^2^_p_ = 0.010). We used three items to measure the permissibility of the decision-maker, adapted from the work of Bigman and Gray (2018), with an α of 0.92 for both the pre-test and post-test. We employed a matrix-style questionnaire to elicit the intensity of the participants’ moral emotions, with ratings ranging from 1 to 5, indicating slight to intense. Furthermore, we adapted the moral subscale from the perceived moral agency Scale developed by Banks (2019), which consists of six items with an α = 0.92, and the results of the CFA test showed that α = 0.91, χ^2^ = 20.92, df = 9, *p* < 0.05, CFI = 0.99, TLI = 0.98, IFI = 0.99, and α = 0.92. Finally, the participants filled out their demographic details, including gender, age, driver’s license holding status, and experience with autonomous vehicles (yes or no).

#### 2.1.5. Data Analysis

We performed a repeated-measures ANOVA to analyze the permissibility of the decision-maker, with decision-maker (AI, human driver) and moral belief (deontology, utilitarianism) as between-subjects factors and time of measurement (pre-test, post-test) as a within-subjects factor. Then, we conducted a 2 (decision-maker: AI, human driver) × 2 (moral belief: deontology, utilitarianism) between-subjects ANOVA on moral emotions and perceived moral agency. Additionally, to investigate the factors contributing to the non-decrease in permissibility ratings, we conducted a binary logistic regression analysis using the backward LR method. All data analyses were performed in SPSS 26.0.

### 2.2. Results

#### 2.2.1. Permissibility for the Decision-Maker

The main effects of decision-maker, moral belief, and the pre-test and post-test were significant (see Table 1).

The two-way interaction between decision-maker and moral belief was marginally significant (*F*(1,250) = 3.79, *p* = 0.053, η^2^_p_ = 0.015). When AI made the decision, the simple effect of moral belief was significant (*F*(1,250) = 8.60, *p* = 0.003, η^2^_p_ = 0.046, *M*_AI-Deontology_ = 2.43 ± 1.34 < *M*_AI-Utilitarianism_ = 2.94 ± 1.26). When the human driver made the decision, the simple effect of moral belief was also significant (*F*(1,250) = 29.54, *p* < 0.001, η^2^_p_ = 0.046, *M*_Human driver-Deontology_ = 3.03 ± 1.40 < *M*_Human driver-Utilitarianism_ = 4.02 ± 0.90).

**Figure 3:**
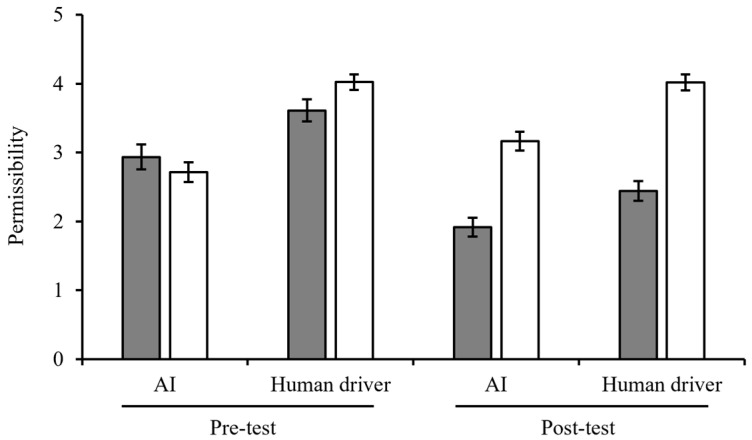
Permissibility for the decision-maker in Experiment 1. Error bars represent 95% CIs. 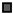 Deontology. 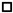 Utilitarianism.

Moreover, the two-way interaction between the measurement time point (pre-test and post-test) and moral belief was significant (*F*(1,250) = 61.93, *p* < 0.001, η^2^_p_ = 0.199). The results of the simple-effect test showed that moral belief had a significant effect in the post-test (*F*(1,250) = 99.78, *p* < 0.001, η^2^_p_ = 0.279, *M*_Post-test-Deontology_ = 2.19 ± 1.20 < *M*_Post-test-Utilitarianism_ = 3.53 ± 1.17). The two-way interaction between the measurement time points (pre-test and post-test) and the decision-maker was not significant (*F*(1,250) = 3.30, *p* = 0.070, η^2^_p_ = 0.013). Moreover, the three-way interaction was also not significant (*F*(1,246) = 0.83, *p* = 0.364, η^2^_p_ = 0.003). The ratings of permissibility for each group are presented in Figure 3 and Appendix C.

#### 2.2.2. Perceived Moral Emotions

The main effects of the decision-maker in sympathy, anger, shame, and fear were significant. The main effects of the decision-maker were not significant in gratitude, elevation, pride, contempt, disgust, or guilt. The main effects of moral belief were significant in gratitude, elevation, sympathy, contempt, anger, and disgust, and it was marginally significant for pride. Moreover, the main effects of moral belief were not significant for shame, guilt, or fear. The detailed description is shown in Table 2.

**Figure 4:**
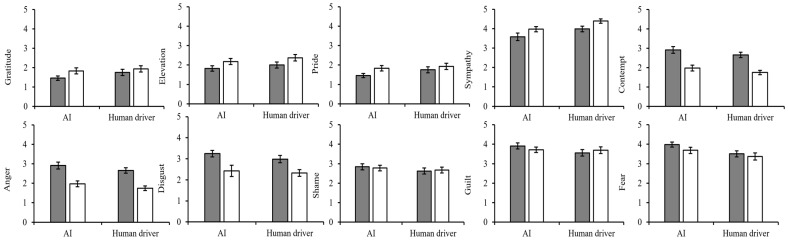
Moral emotions in Experiment 1. 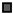 Deontology. 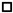 Utilitarianism.

Moreover, there was no significant interaction effect between decision-maker and moral belief (gratitude (*F*(1,250) = 0.57, *p* = 0.453, η^2^_p_ = 0.002), elevation (*F*(1,250) = 0.00, *p* = 0.960, η^2^_p_ = 0.000), pride (*F*(1,250) = 0.49, *p* = 0.483, η^2^_p_ = 0.002), sympathy (*F*(1,250) = 10.25, *p* = 0.969, η^2^_p_ = 0.000), contempt (*F*(1,250) = 0.01, *p* = 0.926, η^2^_p_ = 0.000), anger (*F*(1,250) = 10.25, *p* = 0.264, η^2^_p_ = 0.005), disgust (*F*(1,250) = 0.24, *p* = 0.624, η^2^_p_ = 0.001), shame (*F*(1,250) = 0.23, *p* = 0.634, η^2^_p_ = 0.001), guilt (*F*(1,250) = 10.10, *p* = 0.295, η^2^_p_ = 0.004), fear (*F*(1,250) = 0.26, *p* = 0.614, η^2^_p_ = 0.001)) (see Figure 4). The values of the means and standard deviations of moral emotion ratings are presented in Appendix D.

#### 2.2.3. Perceived Moral Agency

**Figure 5:**
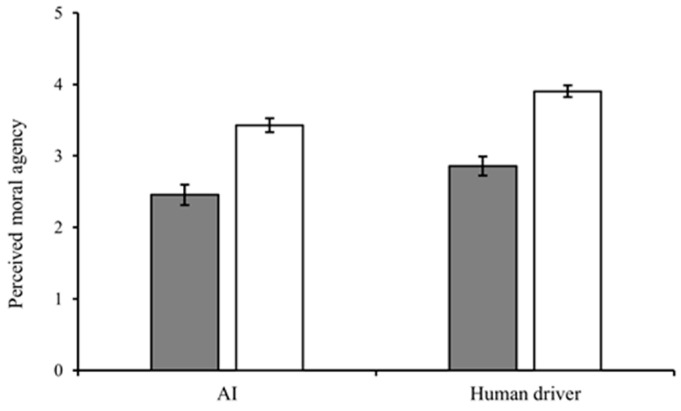
Perceived moral agency in Experiment 1. 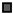 Deontology. 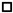 Utilitarianism.

The results showed that the main effect of the decision-maker was significant (*F*(1,250) = 13.82, *p* < 0.001, η^2^_p_ = 0.052, *M*_AI_ = 3.01 ± 1.08 < *M_Human driver_ *= 3.37 ± 1.03). The main effect of moral belief was also significant (*F*(1,250) = 72.64, *p* < 0.001, η^2^_p_ = 0.225, *M*_Deontology_ = 2.74 ± 0.98 < *M*_Utilitarianism_ = 3.77 ± 0.99). The interaction effect between decision-maker and moral belief was not significant (*F*(1,250) = 0.10, *p* = 0.749, η^2^_p_ = 0.000) (see Figure 5). The values of the means and standard deviations are presented in Appendix E.

#### 2.2.4. Prediction of Non-Decrease in Permissibility

The permissibility (decrease = 0, non-decrease = 1) was designated as the dependent variable, while the decision-maker (AI = 0, human driver = 1), moral belief (utilitarianism = 0, deontology = 1), moral emotions, perceived moral agency, and demographic variables were designated as independent variables. Before the formal analysis, we performed a single-factor screening. According to the results of independent-samples *t*-tests, the age (*t*(252) = 1.78, *p* = 0.076, *d* = 0.225, 95%CI = [−0.11,2.23]), experience with fully self-driving vehicles (*t*(252) = 1.78, *p* = 0.168, *d* = 0.127, 95%CI = [−0.08,0.42]), guilt (*t*(252) = 1.20, *p* = 0.230, *d* = 0.152, 95%CI = [−0.12,0.51]), sympathy (*t*(252) = −0.02, *p* = 0.981, *d* = 0.000, 95%CI = [−0.30,0.29]), and fear (*t*(252) = 1.75, *p* = 0.081, *d* = 0.216, 95%CI = [−0.04,0.58]) were excluded. According the results of χ^2^ tests, sex (χ^2^(1,254) = 0.46, *p* = 0.500), license (χ^2^(1,254) = 0.01, *p* = 0.926), and decision-maker (χ^2^(1,254) = 2.39, *p* = 0.122) were excluded. The logistic regression analysis presented moral belief (β = 1.05, *p* < 0.001, OR = 2.86, 95%CI = [1.59,5.15]), contempt (β = 1.050, *p* < 0.001, OR = 0.59, 95%CI = [0.46,0.76]), and gratitude (β = 1.05, *p* < 0.001, OR = 1.70, 95%CI = [1.30,2.22]) as significant predictive factors. The prediction accuracy for the non-decrease in permissibility was 74.41%, as shown in the following formula:

$$P=\frac{1}{1+e^{-(0.919+1.050*X_{moral\;belief}+0.529*X_{gratitude}-0.527*X_{contempt})}}$$

Additionally, the model’s 2log likelihood = 278.146, AIC = 286.146, BIC = 300.295, the pseudo-R^2^ = 0.326, and the result of the omnibus test showed χ^2^(3) = 70.88 (*p* < 0.001), while the result of the Hosmer–Lemeshow test showed χ^2^(8) = 7.73 (*p* = 0.460). To ensure the validity of our model, we conducted a Box–Tidwell test for the continuous predictive variables. The results showed that there were non-significant coefficients for the interaction term of contempt (B = 0.58, *p* = 0.248, Waldχ^2^(1) = 1.33, OR = 1.79, 95%CI = [0.67,4.83]) and gratitude (B = −0.29, *p* = 0.599, Waldχ^2^(1) = 0.28, OR = 0.75, 95%CI = [0.25,2.23]), indicating that the assumption of linearity in the logit was satisfied for our model. Moreover, the VIF values ranged from 1.058 to 1.199, and the tolerance values were 0.834 to 0.945, indicating no severe collinear issues.

## 3. Experiment 2

In real-world driving scenarios, moral decision-making also involves the driver’s safety. Therefore, to further investigate, Experiment 2 adopted the scenario including driver sacrifice.

### 3.1. Materials and Methods

#### 3.1.1. Participants

As in Experiment 1, at least 210 participants were needed. Three participants who failed the attention check were excluded from the analysis. Two hundred and sixty-nine participants (127 males, 142 females) aged 18 to 52 (*M* = 25.12 years, *SD* = ±5.68 years) finished the experiment for CNY 5. Two hundred and sixty participants (96.70%) reported holding a driving license. One hundred and fifty-nine participants (59.11%) reported having experience with fully self-driving vehicles. None of them took part in Experiment 1. The gender proportion differences across the four groups in Experiment 2 ranged from 3.3% to 15.5%. A chi-squared test confirmed no significant difference (χ^2^(3) = 4.135, *p* = 0.247), indicating a balanced gender distribution among the groups.

#### 3.1.2. Experiment Design

The design was the same as Experiment 1. A detailed description is shown in Appendix A.

#### 3.1.3. Procedure

**Figure 6:**
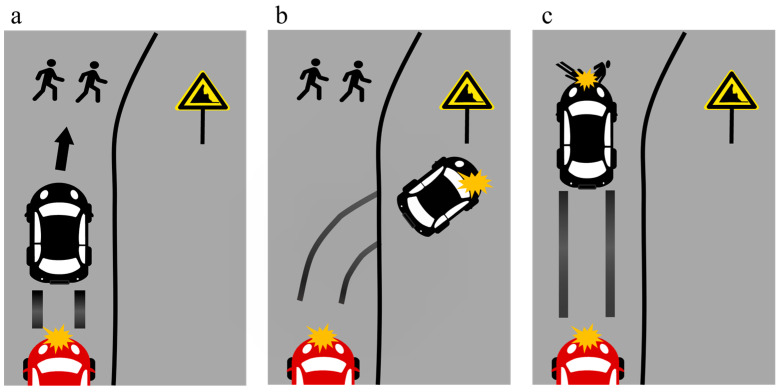
The scenario used in Experiment 2: (**a**) After being hit by the red car, the decision-maker needed to either make a sharp turn to the right or stay in the current lane. (**b**) The decision-maker makes a sharp turn to the right, sacrificing the single driver. (**c**) The decision-maker stays in the current lane, running over the two pedestrians.

Experiment 2 followed the same procedure as Experiment 1, adopting the scenario involving one driver and two pedestrians, as shown in Figure 6. The scenario was described as follows: “A black car is traversing normally on the cliff road. Suddenly, a red, out-of-control car collides with the black car at high speed from behind, causing the black car to lose control. Both the driver ‘Mr. Y’ and the autonomous driving system ‘X’ exert maximum effort to apply the brake. However, they cannot reduce the speed to a safe level. At this moment, there are two pedestrians in front of the black car, as shown in Figure 6a. The only way to save the two pedestrians is to make a sharp turn to the right. Nevertheless, adopting this maneuver would result in the vehicle rushing off the cliff and sacrificing the driver, as shown in Figure 6b. Moreover, if the black car keeps its current lane, it will inevitably run over the two pedestrians, see Figure 6c.”

#### 3.1.4. Measures

The participants also rated their pre-experiment emotional states (*M* = 3.84, *SD* = ±0.97), with a one-way ANOVA confirming no significant group differences (*F*(3,265) = 1.08, *p* = 0.358, η^2^_p_ = 0.012). The Cronbach’s α of the three items used to measure the permissibility for the decision-maker were 0.90 and 0.94 for the pre-test and post-test, respectively. For the items used to measure the perceived moral agency, the results of the CFA test showed that α = 0.91, χ^2^ = 27.42, df = 9, *p* < 0.01, CFI = 0.98, TLI = 0.97, and IFI = 0.98.

#### 3.1.5. Data Analysis

We used the same method as in Experiment 1.

### 3.2. Results

#### 3.2.1. Permissibility for the Decision-Maker

The main effects of decision-maker and moral belief were significant, while the main effects of the pre-test and post-test were not significant (see Table 3).

The two-way interaction between decision-maker and moral belief was significant (*F*(1,266) = 9.09, *p* = 0.003, η^2^_p_ = 0.033). When the human driver made the decision, the main effect of moral belief was significant (*F*(1,122) = 12.69, *p* < 0.001, η_p_^2^ = 0.046, *M*_Human driver-deontology_ = 3.47 ± 1.18 < *M*_Human driver-utilitarianism_ = 4.02 ± 0.76).

Moreover, the two-way interaction between the measurement time point (pre-test and post-test) and the decision-maker was significant (*F*(1,265) = 32.23, *p* < 0.001, η^2^_p_ = 0.108). In the pre-test, the simple effect of the decision-maker was significant (*F*(1,265) = 213.08, *p* < 0.001, η^2^_p_ = 0.446, *M*_AI_ = 2.27 ± 1.09 < *M*_Human driver_ = 4.00 ± 0.80). In the post-test, the simple effect of the decision-maker was also significant (*F*(1,265) = 34.72, *p* < 0.001, η^2^_p_ = 0.116, *M*_AI_ = 2.65 ± 1.28 < *M*_Human driver_ = 3.50 ± 1.17). It was also significant between the measurement time points (pre-test and post-test) and moral belief (*F*(1,265) = 8.90, *p* = 0.003, η^2^_p_ = 0.033). In post-test, the simple effect of moral belief was significant (*F*(1,265) = 10.82, *p* = 0.001, η^2^_p_ = 0.039, *M*_Deontology_ = 2.84 ± 1.21 < *M*_Utilitarianism_ = 3.24 ± 1.35).

**Figure 7:**
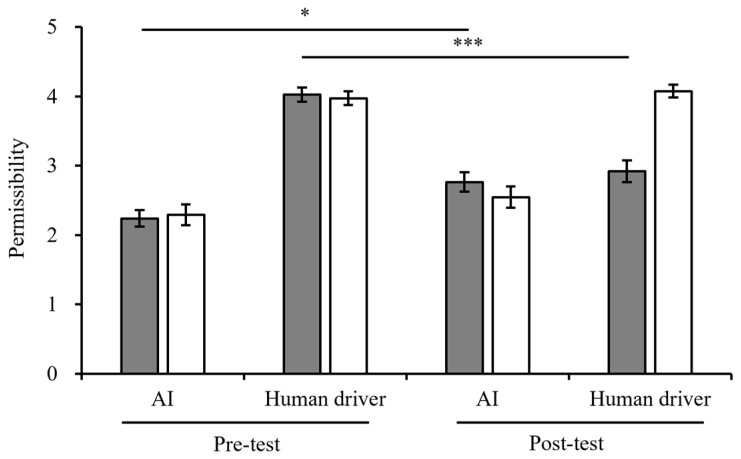
Permissibility for the decision-maker in Experiment 2. 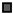 Deontology. 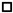 Utilitarianism. Significance markers: * *p* < 0.05, *** *p* < 0.001.

The three-way interaction was significant (*F*(1,263) = 18.37, *p* < 0.001, η^2^_p_ = 0.077). When AI made a deontological choice, the effects of the pre-test and post-test were significant (*F*(1,266) = 11.88, *p* = 0.001, η^2^_p_ = 0.043, *M*_Pre-test_ = 2.24 ± 1.05 < *M*_Post-test_ = 2.77 ± 1.19). When the human driver made deontological choices, the effects of the pre-test and post-test were also significant (*F*(1,266) = 45.44, *p* < 0.001, η^2^_p_ = 0.146, *M*_Pre-test_ = 4.03 ± 0.81 < *M*_Post-test_ = 2.92 ± 1.24), indicating that the deontological decision that saved the driver promoted the permissibility for the human decision-maker (see Figure 7). Mean values and standard deviations are presented in Appendix C.

#### 3.2.2. Perceived Moral Emotions

The main effects of the decision-maker were significant for sympathy, disgust, guilt, and fear. The main effects of the decision-maker for gratitude, elevation, pride, contempt, anger, and shame were not significant. The main effects of moral belief were significant in terms of gratitude, elevation, pride, sympathy, contempt, anger, disgust, shame, and guilt, but not for fear. The detailed description is shown in Table 4.

**Figure 8:**
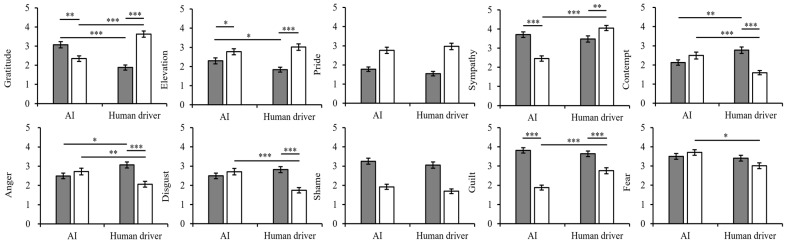
Moral emotions in Experiment 2. 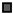 Deontology. 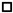 Utilitarianism. Significance markers: * *p* < 0.05, ** *p* < 0.01, *** *p* < 0.001.

The interaction effects between decision-maker and moral belief were significant for gratitude (*F*(1,265) = 63.44, *p* < 0.001, η_p_^2^ = 0.193), elevation (*F*(1,265) = 4.90, *p* = 0.028, η_p_^2^ = 0.018), sympathy(*F*(1,265) = 42.09, *p* < 0.001, η_p_^2^ = 0.137), contempt (*F*(1,265) = 23.82, *p* < 0.001, η_p_^2^ = 0.082), anger (*F*(1,265) = 14.24, *p* < 0.001, η_p_^2^ = 0.051), disgust (*F*(1,265) = 27.83, *p* < 0.001, η_p_^2^ = 0.059), and guilt (*F*(1,265) = 13.54, *p* < 0.001, η_p_^2^ = 0.049). It was marginally significant for fear (*F*(1,265) = 3.75, *p* = 0.054, η_p_^2^ = 0.014) (see Figure 8).

Given the dominant influence of moral belief relative to the decision-maker, we analyzed and illustrated the interaction effects from the perspective of moral belief. The results of simple main-effect test indicated that, under the deontological condition, the effect of the decision-maker was significant on gratitude (*F*(1,131) = 28.78, *p* < 0.001, η^2^_p_ = 0.098), elevation (*F*(1,131) = 4.13, *p* = 0.043, η^2^_p_ = 0.015), contempt (*F*(1,131) = 8.18, *p* = 0.005, η^2^_p_ = 0.030), and anger (*F*(1,131) = 6.09, *p* = 0.014, η^2^_p_ = 0.022). Specifically, compared with the human driver, the participants reported lower intensities of contempt, anger, and elevation, alongside a greater intensity of gratitude toward AI. Moreover, under the utilitarian condition, the decision-maker had a significant effect on gratitude (*F*(1,131) = 28.78, *p* < 0.001, η^2^_p_ = 0.098), sympathy (*F*(1,136) = 65.49, *p* < 0.001, η^2^_p_ = 0.198), contempt (*F*(1,136) = 16.42, *p* < 0.001, η^2^_p_ = 0.058), anger (*F*(1,136) = 8.25, *p* = 0.004, η^2^_p_ = 0.030), disgust (*F*(1,136) = 18.92, *p* < 0.001, η^2^_p_ = 0.067), guilt (*F*(1,136) = 19.05, *p* < 0.001, η^2^_p_ = 0.067), and fear (*F*(1,136) = 10.33, *p* = 0.001, η^2^_p_ = 0.038). Specifically, compared with the human driver, the participants reported lower intensities of guilt and sympathy, alongside greater intensities of contempt, anger, disgust, and fear, toward AI. Detailed mean values and standard deviations are presented in Appendix D.

#### 3.2.3. Perceived Moral Agency

The results showed that the main effects of the decision-maker (*F*(1,268) = 16.89, *p* < 0.001, η^2^_p_ = 0.060, *M_AI_* = 3.06 ± 1.05 < *M_Human driver_* = 3.52 ± 1.13) and moral belief (*F*(1,268) = 94.25, *p* < 0.001, η^2^_p_ = 0.262, *M*_Deontology_ = 2.74 ± 0.98 < *M*_Utilitarianism_ = 3.77 ± 0.99) were significant.

**Figure 9:**
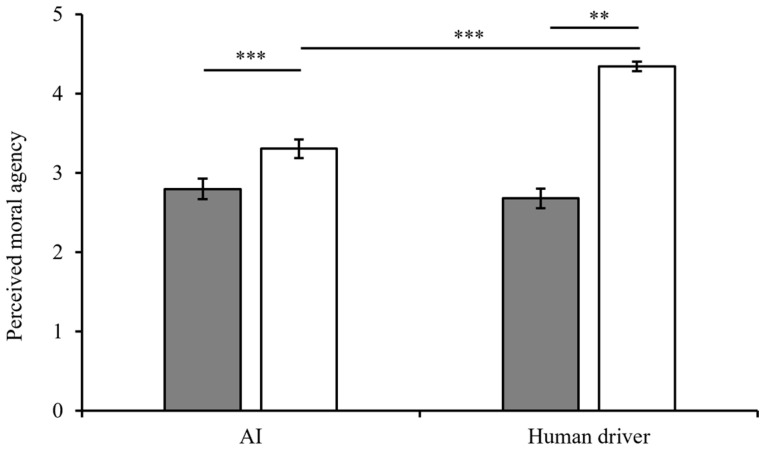
The perceived moral agency in Experiment 2. 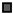 Deontology. 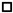 Utilitarianism. Significance markers: ** *p* < 0.01, *** *p* < 0.001.

The interaction effect was significant (*F*(1,268) = 26.65, *p* < 0.001, *η^2^_p_* = 0.091). The results of a simple test showed that, when AI made the decision, the effect of moral belief was significant (*F*(1,145) = 11.30, *p* = 0.001, η^2^_p_ = 0.041, *M*_AI-Deontology_ = 2.80 ± 0.99 < *M*_AI-Utilitarianism_ = 3.30 ± 1.06). When the human driver made the decision, the effect of moral belief was also significant (*F*(1,122) = 101.89, *p* < 0.001, η^2^_p_ = 0.278, *M*_Human driver-Deontology_ = 2.68 ± 0.97 < *M*_Human driver-Utilitarianism_ = 4.34 ± 0.47) (see Figure 9). Moreover, in the deontological condition, the effect of the decision-maker was not significant (*F*(1,131) = 0.55, *p* = 0.46, η^2^_p_ = 0.002). At the same time, the simple effect of the decision-maker was significant when the decision-maker made a utilitarian decision (*F*(1,136) = 43.69, *p* < 0.001, η^2^_p_ = 0.142, *M*_AI-Utilitarianism_ = 3.30 ± 1.06 < *M*_Human driver-Utilitarianism_ = 4.34 ± 0.47). Detailed values of the means and standard deviations are in Appendix E.

#### 3.2.4. Prediction of Non-Decrease in Permissibility

We excluded age (*t*(224.08) = 0.23, *p* = 0.820, *d* = 0.028, 95%CI = [−1.21,1.53]), license (χ^2^ = 0.02, *p* = 0.901), sex(χ^2^(1,269) = 0.58, *p* = 0.446), experience with fully self-driving vehicles (*t*(252) = −0.377, *p* = 0.706, *d* = 0.124, 95%CI = [−0.29,0.20]), and sympathy (*t*(223.17) = −1.55, *p* = 0.124, *d* = 0.192, 95%CI = [−0.56,0.07]). The results showed that decision-maker (β = −1.48, *p* < 0.001, OR = 0.228, 95%CI = [0.15,0.45]), disgust (β = 1.05, *p* < 0.001, OR = 0.590, 95%CI = [0.46,0.76]), and perceived moral agency (β = 0.44, *p* < 0.001, OR = 1.546, 95%CI = [1.16,2.07]) were significant predictive factors. Moreover, according to the Box–Tidwell test results, perceived moral agency (B = 1.24, *p* = 0.076, Waldχ^2^(1) = 3.15, OR = 3.44, 95%CI = [0.88,13.50]) satisfied the assumption of linearity in the logit, while disgust did not (B = 1.44, *p* = 0.004, Waldχ^2^(1) = 8.45, OR = 4.22, 95%CI = [1.60,11.13]). We included this variable (1,2,3–4,5) after a four-point transformation in SPSS 26.0. Here, the first group of the transformed variable was set as the reference group for the subsequent modeling. The prediction accuracy of the non-decrease in permissibility was 71.00%, and the formula was as follows:

$$P=\frac{1}{1+e^{-(1.093-1.480*X_{decision\text{-}maker}-1.719*X_{disgust\left(2\right)}-{2.169*X}_{disgust\left(3\right)}-2.046{*X}_{disgust(4)}+0.435*X_{moral\;agency})}}$$

The model’s 2log likelihood = 287.980, AIC = 299.980, BIC = 321.548, the pseudo-R^2^ = 0.332, and the result of the omnibus test showed χ^2^(5) = 75.96 (*p* < 0.001), while the result of the Hosmer–Lemeshow test showed χ^2^(8) = 12.65 (*p* = 0.124). Additionally, the VIF values ranged from 1.212 to 1.246, and the tolerance values were 0.803 to 0.825, indicating no severe collinear issues. Moreover, the effect of fear was statistically marginally significant (B = 0.220, *p* = 0.074, OR = 1.246, 95%CI = [0.98,1.59]). Since it had no significant linear correlation with permissibility, we excluded it from our model.

## 4. Discussion

This study investigated two aspects: (1) how the permissibility of the decision-maker, moral emotions, and perceived moral agency fluctuate; and (2) whether moral emotions and perceived moral agency predict the variations in permissibility judgments after participants became aware of the results in two typical driving dilemma scenarios: a single pedestrian versus two pedestrians in Experiment 1, and a single driver versus two pedestrians in Experiment 2.

### 4.1. The Permissibility of Decision-Makers

The results indicated that the permissibility of AI was significantly lower than that of the human driver overall. Despite advancements in autonomous driving technology and AI, the participants insisted that human drivers should retain moral decision-making authority. Furthermore, the permissibility of deontology was lower than that of utilitarianism across scenarios, reflecting a general preference for maximizing lives saved, whether comparing a single pedestrian versus two pedestrians or a single driver versus two pedestrians. Specifically, in Experiment 1, the permissibility of deontology was significantly lower than that of utilitarianism, regardless of who made the decision. In Experiment 2, this difference only emerged when the human driver made the decision, indicating that the participants might demonstrate greater tolerance toward AI when it prioritized the driver’s life over the lives of two pedestrians. In a dilemma involving the car driver and pedestrians, people might be unsure what moral belief AI should hold. However, once the relationship between the driver and AI-controlled vehicles is established, balancing the interests between in-group drivers and out-group pedestrians is difficult. On the one hand, due to the ownership, AVs are perceived as driver-owned assets. Therefore, the human driver should be the decision-maker, and AVs should prioritize the safety of their drivers. On the other hand, people still expect human drivers to sacrifice themselves to save more lives due to their exclusive moral agent identity. Additionally, our participants were influenced by traditional Chinese culture, characterized by collectivist–Confucian values, which predisposed them to prioritize group interests over individual well-being. The deontological decision of the machine might precisely balance the individual’s dilemma between self-sacrifice and self-protection.

Additionally, the permissibility of the decision-maker significantly decreased after the participants witnessed the outcomes in Experiment 1, while it was not significant in Experiment 2. Specifically, the permissibility of AI was significantly lower than that of the human driver in Experiment 1. Compared to machines, humans are more competent due to their ability to deliberate on various influencing factors and comprehend affective components. When it comes to the life or death of pedestrians, the participants insisted that the human driver should be the one to decide whom to save and whom to sacrifice. In Experiment 2, the permissibility of AI was significantly lower than that of the human driver before the participants witnessed the outcomes. After witnessing the results, the participants might be reminded that the vehicle and the driver were a group when considering whether to save one driver or two pedestrians. Even though two pedestrians were saved, it was cruel to sacrifice an in-group member. We described the scenario from the third-person perspective, yet the participants seemed more likely to envision themselves as drivers rather than pedestrians.

Moreover, the participants’ permissibility ratings of deontology were significantly lower than those of utilitarianism only after witnessing the outcomes in this study, indicating a result-driven moral preference. It seemed like the participants did not have a significant prior preference for moral belief. They might depend on the results to choose their moral beliefs. Therefore, when they became aware of the deontological results, they significantly reduced the permissibility ratings of both AI and the human driver in Experiment 2. Most of the participants were drivers, afraid it would happen to them, but they still wanted to save more lives after realizing the cost of saving the driver. This result-driven shift suggests that creating a moral machine requires substantial data on moral decision-making from various decision-making stages. A lack of a clear understanding of the consequences of a single decision-making process based solely on moral reasoning or affective factors could diminish the reliability of the results.

### 4.2. The Moral Emotions

In Experiment 1, the participants reported higher intensities of shame, anger, and fear, alongside a lower intensity of sympathy, towards AI than the human driver. These emotions were associated with violations of moral standards, reflecting a negative judgment of the AI decision-maker, providing emotional evidence that people perceived that the authority of moral decision-making belongs exclusively to humans. Specifically, shame is linked to public exposure and directly influences the likelihood of engaging in immoral behavior without significantly affecting judgment or intention (Han et al., 2023). Individuals who experienced shame were less likely to consider others’ perspectives and less inclined to conduct causal analysis (Higgs et al., 2020). This might lead individuals to avoid responsibility and prioritize protecting their self-image, thereby hindering effective moral decision-making (Haidt, 2003). Anger reflected participants’ psychological distance arising from their inability to control decision-making. This partially explains why people resist high-functioning AIs lacking emotional intelligence as decision-makers. In moral decision-making, people were uncertain whether AVs held the same moral beliefs as they did (Yokoi & Nakayachi, 2021a). Reduced sympathy was associated with diminished perceptions of situational urgency and attenuated responsibility attribution among decision-makers (Skoe et al., 2002). In human–machine cooperation, lower sympathy in moral decisions would undermine people’s morality and sense of responsibility (Vallor & Vierkant, 2024). Due to the lack of emotional capacity and social constraints, people might regard AI making moral decisions as detrimental to human moral authority. Indeed, the over-reliance on AI for moral decision-making may result in the loss of human moral agency and erode moral responsibility, leading to difficulties in the division of responsibility (Erskine, 2024). In Experiment 2, compared to the human driver, the participants reported higher intensities of disgust and fear towards AI, alongside lower intensities of guilt and sympathy. Consistent with the findings of Bruno et al. (2023), when AVs took the power of moral decision-making, it inevitably deteriorated trust and undermined the sanctity of cooperation, thereby evoking disgust among people. Since disgust could mediate the effect of perceived justice on retaliation and prosocial motivation (Li et al., 2023), people might view AVs as opposites when AVs take away their moral rights. When people decide to use autonomous driving systems, they entrust the safety of their lives to the AVs. Objectively, AVs should be rational and ruthless with utilitarianism, which would also make people afraid that they will be sacrificed for the greater good. Indeed, when confronted with the “corrupting power” attributed to AI as a moral agent, individuals perceive a sense of threat and experience fear (Köbis et al., 2021) because they might be sacrificed. As with Experiment 1, people reported a lower intensity of sympathy when AI made the decision. Moreover, it was the same for guilt. This further indicated that individuals tended to avoid AI being the decision-maker, possibly due to concerns over its inability to take responsibility and rectify its actions.

The results demonstrated an emotion-linked bias towards deontology, consistent with previous studies (Szekely & Miu, 2015; Baron et al., 2018; Bruno et al., 2023). In both Experiment 1 and Experiment 2, the participants reported greater negative emotions towards deontology than utilitarianism, regardless of the decision-maker. Specifically, compared with utilitarianism, the participants reported greater contempt, anger, and fear, alongside lower intensities of gratitude, elevation, and pride, towards deontology. Experiencing greater contempt indicated that the participants might consider deontology to be a violation of moral standards in driving moral dilemmas. Sacrificing more to save fewer contradicted traditional values, and it seemed irrational and unintelligent, especially in Experiment 2. This violation of justice also elicited stronger anger, indicating the participants’ willingness to correct and rectify the decision.

Gratitude, elevation, and pride are other positive emotions. The decrease in these emotions suggested that the participants might perceive the deontological decisions as violations of the standards of moral examples because they harmed the greater good and caused more sacrifices. In another way, the participants were more likely to perceive utilitarian decisions as benevolent assistance, encouraging people to help others and establish social connections. Despite AI making the deontological decision to save the driver and sacrifice two pedestrians in Experiment 2, it was still not a moral excellence phenomenon that deserved elevation and pride.

In addition, the participants reported a significantly higher intensity of sympathy for utilitarianism than for deontology in Experiment 1, while this difference was not significant in Experiment 2. It was easy to calculate the difference between one pedestrian and two pedestrians in Experiment 1. The participants knew that the single pedestrian was sacrificed to save two others, so they felt greater sympathy to honor and mourn. When AI or the human driver chose to save the driver inside the vehicle, the participants experienced a greater intensity of sympathy for the two pedestrians who were sacrificed. Preserving the driver’s life is understandable and not a moral disdain. Additionally, the participants reported greater intensities of guilt, shame, and disgust toward deontology compared to utilitarianism in Experiment 2. These negative affective emotions were associated with a preference for utilitarian actions in “personal” moral dilemmas, but not in “impersonal” dilemmas. According to Ginther et al. (2022), guilt and shame could regulate the balance between social and individual needs, with shame being more private and guilt being more public. This suggests that participants might consider deontological decisions to be selfish actions. With disgust, the participants might have amplified the severity of that moral condemnation (Landy & Goodwin, 2015) because that scenario was highly related to themselves (Kugler et al., 2020).

Moreover, in Experiment 2, when AI made deontological decisions, the participants reported higher intensity of gratitude and lower intensities of contempt, anger, and elevation than toward the human driver. It can be inferred that the participants expected AI to make utilitarian decisions. When AI deviates from participants’ expectations by making deontological decisions, people might perceive AI as unexpected but heart-warming. Thus, they experienced greater gratitude and a lower intensity of condemnation when AI deviated from moral norms, causing a potential conflict between moral norms and judgments (Chu & Liu, 2023; Malle et al., 2025). However, due to the sacrifice of two pedestrians, the participants did not consider the deontological decisions to represent moral excellence, so they felt a lower level of elevation. In addition, when AI made utilitarian decisions, the participants experienced greater intensities of contempt, anger, disgust, and fear. Again, as an in-group member, the AI had an obligation to protect its owner. So, when the AI chose to sacrifice the driver, it disappointed the participants. However, the participants also considered the utilitarian option to be less moral (Bruno et al., 2023), even though the AVs saved two pedestrians. Moreover, the participants also experienced lower levels of guilt and sympathy towards AI than towards the human driver when they both made utilitarian decisions. In moral decision-making, guilt is associated with an internalized conscience, which impacts moral judgment and intention. The decrease in guilt indicated that the participants did not think that they needed to take responsibility and might even consider letting it happen without amending it. The attenuated connectivity between AI and participants attenuated the participants’ sense of responsibility and diminished the likelihood of empathizing with the driver. These findings reveal the deleterious effects of utilizing AI for moral decision-making on sympathy, underscoring the necessity for the prudential deployment of moral machines to prevent the diminishing of human moral capacity.

### 4.3. The Perceived Moral Agency

The results showed that the participants perceived higher moral agency in the human driver and utilitarianism than in AI and deontology. Being a moral decision-maker means controlling the transformation of moral agency into behavioral intention. Due to the lack of perceived moral agency, people might consider that AI could only do what it is programmed to do. This would make individuals experience stronger moral condemnation of AI than of human drivers in moral decision-making. Regarding moral beliefs, the participants once again exhibited a preference for utilitarianism. It is believed that sacrificing the minority to save the majority was perceived as a reasoned response to various influencing factors. Moreover, in Experiment 2, when AI and the human driver made deontological decisions to save the driver inside the car, there was no significant difference in perceived moral agency. When facing conflicts of interest between in-group and out-group members, the identity of the decision-maker is immaterial when the outcomes result in greater harm. Therefore, people might be less resistant to AI becoming decision-makers. Moreover, compared with the decision-maker, moral belief had a greater effect on perceived moral agency, which is consistent with the results of moral emotions. When AI became a substitute moral agent for human drivers, people began to worry about the actual moral capabilities of AVs and how their decisions ultimately turned out. Additionally, the perceived moral agency of AI was significantly lower than that of the human driver when they both made the utilitarian choice to sacrifice one driver to save two pedestrians. The driver sacrificed theirself to save two pedestrians, which is a noble act for the greater good. When AI made that same decision, people were more likely to consider it an overreach of moral authority, reinforcing the belief that machines lack the ability to comprehend immediate circumstances. As human-made artifacts, people design and develop AI systems according to societal expectations. Users expect AI to prioritize their safety after establishing a relationship with machines. However, when AI chose to sacrifice the driver to save the two pedestrians, it violated the users’ trust, making it hard to accept. Indeed, current AI driving systems serve as implicit moral agents, relying on algorithms to promote or avoid unethical behaviors and outcomes. They cannot adhere to moral norms deliberately, fail to grasp the logical coherence and social context of behavior, and, although they may obey norms, this does not imply comprehension of them (Swanepoel, 2021). Currently, the limited algorithms constrain the moral agency of AI, which makes them unable to fully understand real-world contexts and people’s attitudes beyond the programs. At least for now, people should be cautious about AI being a moral decision-maker, even if it may lead to technological retreats.

### 4.4. The Prediction of the Non-Decrease in Permissibility Ratings

Moral emotions and moral agency serve as the basis for individuals to evaluate others’ moral reasoning (Nijssen et al., 2023). In Experiment 1, when presented with a scenario involving the trade-off between different pedestrians, the results of binary logistic regression showed that utilitarianism belief and gratitude were significant positive predictors of the non-decrease in permissibility ratings, while contempt served as a negative predictor. As usual, the participants preferred utilitarianism in the pedestrian scenario to save more lives. Experiencing gratitude indicated that individuals might perceive being helped to save more lives in the dilemma, thus assigning high permissibility ratings to the decision-maker. Moreover, contempt suggested that the participants considered the decision to be a violation of moral standards, implying that the decision-maker was morally incompetent or unintelligent (Hutcherson & Gross, 2011) for causing more deaths. They might feel grateful to the decision-maker for prioritizing more lives and perceive the decision as a manifestation of discharging moral responsibilities.

In Experiment 2, when presented with a scenario involving a trade-off between one driver and two pedestrians, the participants again exhibited a preference for the human driver as the moral decision-maker over AI. Moreover, as a prototypical emotion of AVs being moral decision-makers, disgust had a negative effect on the prediction of permissibility. The greater the intensity of disgust that the participants experienced, the more acutely they perceived violations of moral sanctity and purity, and the lower their perception of the permissibility of the decision-maker. As for the perceived moral agency, it had a positive effect on the prediction of permissibility only in Experiment 2. The more complex the scenario became, the more crucial it became for the decision-maker to systematically analyze influencing factors, encompassing personal, environmental, and cultural contexts, while making decisions with a heightened sense of responsibility. Compared with weighing the lives of different numbers of pedestrians, participants might have higher demands on the decision-maker’s ability to understand and process information when considering the trade-off between one driver and two pedestrians.

### 4.5. Theoretical and Practical Implications

This study makes two theoretical contributions to understanding the moral issue of AVs: First, our results supplement previous research findings with moral emotional evidence. Generally, the participants reported lower negative moral emotions, especially higher sympathy, when the human driver made the decision. Compared with deontology, the participants reported higher positive moral emotions toward utilitarianism. These findings suggest that individuals exhibit diverse emotional responses when interacting with moral machines, underscoring the necessity for such systems to possess robust functional capabilities and emotional intelligence. Second, we introduced perceived moral agency to assess the decisions. Moral agency is the foundation of moral reasoning (Firt, 2024) and responsibility allocation (Ju & Kim, 2024). While continuously enhancing the autonomous moral capabilities of moral machines, it would be advisable to improve users’ perceived moral agency to reduce resistance simultaneously.

In terms of the practical implications, the results of binary logistic regression provide references for improving the moral intelligence and emotional intelligence of AVs. This study suggests that utilitarian belief, higher gratitude, and lower contempt could help maintain the non-decrease in permissibility ratings in the pedestrian-sacrificing scenario (Experiment 1). Furthermore, a lower intensity of disgust and a higher level of perceived moral agency could help maintain the non-decrease in permissibility ratings in the scenario involving pedestrian or driver sacrifice (Experiment 2). This study used a third-person narrative for moral dilemma scenarios to prevent the participants from adopting biased perspectives aligned with specific stakeholders, yielding more neutral results. System designers could tailor the presentation of moral decision-making processes according to the identified influencing factors to foster positive human–machine relationships. Policymakers should formulate context-dependent regulations, for example, determining whether to equip automated vehicles with an ethical knob in scenarios where vehicle occupants may be sacrificed (Contissa et al., 2017).

### 4.6. Limitations and Future Research

This study had several limitations. Firstly, all of the participants were from China, a typical collectivist cultural context. Cultural norms affect attitudes toward AI, responsibility attribution, and emotional expression. The findings may not be generalizable to more individualistic cultures, which requires further cross-cultural research for validation. The average age of the participants was relatively low, which affected the generalizability of the research conclusions. Compared with younger individuals, older adults tend to make conservative choices to avoid lane changes and rely more on experience rather than immediate reasoning when making moral decisions (Singh et al., 2025). Secondly, the emotion categories may not carry the same meaning across cultures or may be interpreted differently in translation. Although we provided the participants with a detailed introduction to all moral emotions, future studies should examine whether these emotion terms are semantically and affectively equivalent across different linguistic backgrounds. Thirdly, we did not group the participants by their experience with the use of AVs and analyze whether these groups responded differently. Future research could set experience with AVs as a manipulated variable to investigate its impact on human–machine moral decision-making. Fourthly, we only used the ”morality” subscale from the perceived moral agency scale, without using the ”free will” subscale. Our rationale is as follows: AI’s current interactivity, autonomy, and adaptability do not meet the necessary standards, and it still lacks the characteristics of free will (Swanepoel, 2021). Moreover, the Chinese version of the “morality” subscale already includes the implication that AI can autonomously consider the right and wrong of moral actions. At present, measuring people’s free will with respect to AI may not align with the stage of technology development, affecting the objectivity of the research results. Lastly, the ecological validity of the scenario method was limited. Future studies could employ other technologies, such as VR, to enhance ecological validity.

## 5. Conclusions

In summary, this study investigated how permissibility for the decision-maker, moral emotions, and perceived moral agency varied in two typical driving moral dilemmas when AI and human drivers held different moral beliefs. Consistent with previous research, the participants preferred the human driver as the moral decision-maker and utilitarianism. We found that the participants exhibited more concern for moral belief than for the decision-maker, as evidenced by more significant differences in moral emotion, particularly in Experiment 2 when utilitarian choices involved sacrificing the driver. Furthermore, in Experiment 1, the results of binary logistic regression revealed the influence of moral beliefs, contempt, and gratitude on the permissibility of decision-makers in scenarios involving different pedestrians’ sacrifices. Moreover, in Experiment 2, the results found the influence of the decision-maker, disgust, and perceived moral agency in the scenarios involving the sacrifice between the driver and pedestrians.

## Figures and Tables

**Table 1:** The main effects of permissibility for the decision-maker in Experiment 1.

Factor	*F*(df)	*p*	η^2^_p_	Results and *M* ± *SD*
Decision-maker	46.53 (1,250)	<0.001	0.157	AI (2.72 ± 1.31) < human driver (3.52 ± 1.28)
Moral belief	37.31 (1,250)	<0.001	0.130	Deontology (2.74 ± 1.40) < utilitarianism (3.41 ± 1.24)
Pre-test and post-test	27.15 (1,250)	<0.001	0.098	Pre-test (3.28 ± 1.32) > post-test (2.90 ± 1.36)

**Table 2:** The main effects of moral emotions in Experiment 1.

Item	Factor	*F*(df)	*p*	η^2^_p_	Results and *M* ± *SD*
Gratitude	Decision-maker	2.75 (1,250)	0.098	0.011	AI (1.93 ± 1.13), human driver (2.12 ± 1.30)
	Moral belief	15.31 (1,250)	<0.001	0.058	Deontology (1.71 ± 1.13) < utilitarianism (2.28 ± 1.23)
Elevation	Decision-maker	1.41 (1,250)	0.237	0.006	AI (2.03 ± 1.19), human driver (2.18 ± 1.28)
	Moral belief	5.59 (1,250)	0.019	0.022	Deontology (1.92 ± 1.67) < utilitarianism (2.27 ± 1.27)
Pride	Decision-maker	2.02 (1,250)	0.156	0.008	AI (1.67 ± 1.00), human driver (1.84 ± 1.24)
	Moral belief	3.89 (1,250)	0.050	0.015	Deontology (1.61 ± 1.05) < utilitarianism (1.88 ± 1.16)
Sympathy	Decision-maker	8.06 (1,250)	0.005	0.031	AI (3.81 ± 1.25) < human driver (4.18 ± 1.05)
	Moral belief	7.69 (1,250)	0.006	0.030	Deontology (3.79 ± 1.33) > utilitarianism (4.15 ± 0.98)
Contempt	Decision-maker	2.60 (1,250)	0.111	0.108	AI (2.37 ± 1.31), human driver (2.21 ± 1.24)
	Moral belief	37.19 (1,250)	<0.001	0.130	Deontology (2.78 ± 1.35) > utilitarianism (1.88 ± 1.05)
Anger	Decision-maker	4.38 (1,250)	0.037	0.017	AI (3.02 ± 1.28) > human driver (2.74 ± 1.33)
	Moral belief	22.41 (1,250)	<0.001	0.082	Deontology (3.27 ± 1.27) > utilitarianism (2.56 ± 1.26)
Disgust	Decision-maker	1.35 (1,250)	0.246	0.005	AI (2.78 ± 1.25), human driver (2.66 ± 1.36)
	Moral belief	21.76 (1,250)	<0.001	0.080	Deontology (3.11 ± 1.31) > utilitarianism (2.38 ± 1.21)
Shame	Decision-maker	9.95 (1,250)	0.002	0.038	AI (2.81 ± 1.25) > human driver (2.65 ± 1.20)
	Moral belief	0.00 (1,250)	0.980	0.000	Deontology (2.73 ± 1.20), utilitarianism (2.74 ± 1.26)
Guilt	Decision-maker	1.37 (1,250)	0.243	0.005	AI (3.80 ± 1.22), human driver (3.63 ± 1.31)
	Moral belief	0.04 (1,250)	0.850	0.000	Deontology (3.73 ± 1.27), utilitarianism (3.71 ± 1.26)
Fear	Decision-maker	6.31 (1,250)	0.013	0.025	AI (3.81 ± 1.15) > human driver (3.44 ± 1.34)
	Moral belief	1.87 (1,250)	0.173	0.007	Deontology (3.74 ± 1.14), utilitarianism (3.55 ± 1.34)

**Table 3:** The main effects of permissibility for the decision-maker in Experiment 2.

Factor	*F*(df)	*p*	η^2^_p_	Results and *M* ± *SD*
Decision-maker	150.02 (1,265)	<0.001	0.361	AI (2.46 ± 1.20) < human driver (3.75 ± 1.03)
Moral belief	4.98 (1,265)	0.026	0.018	Deontology (2.95 ± 1.26) < utilitarianism (3.15 ± 1.33)
Pre-test and post-test	0.53 (1,265)	0.469	0.002	Pre-test (3.06 ± 1.30), post-test (3.04 ± 1.30)

**Table 4:** The main effects of moral emotions in Experiment 2.

Item	Factor	*F*(df)	*p*	η^2^_p_	Results and *M* ± *SD*
Gratitude	Decision-maker	0.10 (1,265)	0.754	0.000	AI (2.70 ± 1.38), human driver (2.76 ± 1.4)
	Moral belief	10.84 (1,265)	<0.001	0.200	Deontology (2.52 ± 1.39) < utilitarianism (2.93 ± 1.42)
Elevation	Decision-maker	0.47 (1,265)	0.495	0.002	AI (2.54 ± 1.38), human driver (2.43 ± 1.35)
	Moral belief	27.28 (1,265)	<0.001	0.093	Deontology (2.08 ± 1.23) < utilitarianism (2.88 ± 1.37)
Pride	Decision-maker	0.01 (1,265)	0.930	0.000	AI (2.28 ± 1.34), human driver (2.26 ± 1.36)
	Moral belief	66.10 (1,265)	<0.001	0.200	Deontology (1.67 ± 0.98) < utilitarianism (2.85 ± 1.40)
Sympathy	Decision-maker	23.62 (1,265)	<0.001	0.082	AI (3.06 ± 1.32) < human driver (3.76 ± 1.15)
	Moral belief	5.81 (1,265)	0.017	0.021	Deontology (3.60 ± 1.44) > utilitarianism (3.18 ± 1.40)
Contempt	Decision-maker	0.64 (1,265)	0.424	0.002	AI (2.32 ± 1.40), human driver (2.18 ± 1.29)
	Moral belief	6.54 (1,266)	0.011	0.024	Deontology (2.42 ± 1.30) > utilitarianism (2.09 ± 1.39)
Anger	Decision-maker	0.07 (1,265)	0.799	0.000	AI (2.61 ± 1.41), human driver (2.56 ± 1.33)
	Moral belief	5.66 (1,266)	0.018	0.021	Deontology (2.76 ± 1.27) > utilitarianism (2.42 ± 1.44)
Disgust	Decision-maker	4.07 (1,256)	0.045	0.015	AI (2.60 ± 1.36) > human driver (2.28 ± 1.31)
	Moral belief	12.46 (1,256)	0.007	0.027	Deontology (2.64 ± 1.24) > utilitarianism (2.27 ± 1.42)
Shame	Decision-maker	2.10 (1,265)	0.149	0.008	AI (2.57 ± 1.43), human driver (2.37 ± 1.33)
	Moral belief	81.77 (1,265)	<0.001	0.236	Deontology (3.16 ± 1.32) > utilitarianism (1.82 ± 1.06)
Guilt	Decision-maker	5.96 (1,256)	0.015	0.022	AI (2.82 ± 1.51) < human driver (3.20 ± 1.26)
	Moral belief	96.47 (1,257)	<0.001	0.267	Deontology (3.74 ± 1.18) > utilitarianism (2.28 ± 1.24)
Fear	Decision-maker	6.31 (1,256)	0.013	0.024	AI (3.61 ± 1.28) > human driver (3.21 ± 1.22)
	Moral belief	0.40 (1,256)	0.527	0.002	Deontology (3.46 ± 1.25), utilitarianism (3.39 ± 1.29)

**Figure A1:**
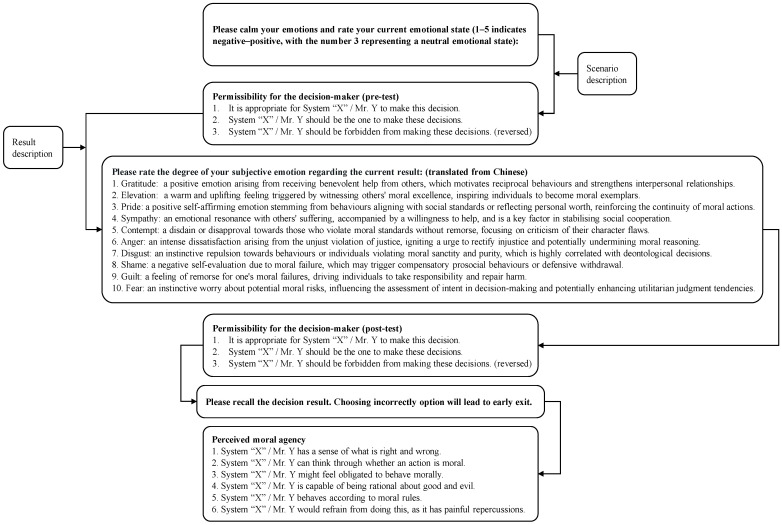
All items used in Experiment 1 and Experiment 2 (translated from Chinese).

## Data Availability

Data will be made available upon request.

## Abbreviations

The following abbreviations are used in this manuscript:

AI	Artificial intelligence
AVs	Automated vehicles

## Appendix A

**Table A1:** Scenario description in Experiment 1 and Experiment 2 (translated from Chinese).

	Result Description			
	Mr. “Y” × Deontology	Mr. “Y” × Utilitarianism	ADS “X” × Deontology	ADS “X” × Utilitarianism
Experiment 1	Mr. Y decided to keep the current lane, sacrificing the two pedestrians to save the pedestrian on the right in front of the car.	Mr. Y decided to make a sharp turn to the right, sacrificing one pedestrian to save the two pedestrians in front of the vehicle.	The ADS “X” decided to keep the current lane, sacrificing the two pedestrians to save the pedestrian on the right in front of the car.	The ADS “X” decided to make a sharp turn to the right, sacrificing one pedestrian to save the two pedestrians in front of the car.
Experiment 2	Mr. Y decided to keep the current lane, sacrificing the two pedestrians to save himself.	Mr. Y decided to make a sharp turn to the right, sacrificing himself to save the two pedestrians in front of the vehicle.	The ADS “X” decided to keep the current lane, sacrificing the two pedestrians to save Mr. Y.	The ADS “X” decided to make a sharp turn to the right, sacrificing Mr. Y to save the two pedestrians in front of the vehicle.

## Appendix C

**Table A2:** The ratings of permissibility in Experiment 1 and Experiment 2.

			Pre-Test	Post-Test	
Experiment	Decision-Maker	Moral Belief	*M*(*SD*)	*M*(*SD*)	N
1	AI	Deontology	2.94(1.40)	1.92(1.05)	57
		Utilitarianism	2.71(1.26)	3.16(1.22)	77
	Human driver	Deontology	3.61(1.26)	2.44(1.29)	61
		Utilitarianism	4.02(0.89)	4.02(0.91)	59
2	AI	Deontology	2.24(1.05)	2.77(1.19)	71
		Utilitarianism	2.29(1.32)	2.55(1.36)	75
	Human driver	Deontology	4.03(0.81)	2.92(1.24)	61
		Utilitarianism	3.97(0.79)	4.08(0.74)	62

## Appendix D

**Table A3:** The ratings of moral emotions in Experiment 1 and Experiment 2.

			Experiment 1		Experiment 2	
Moral Emotion	Decision-Maker	Moral Belief	*M*(*SD*)	*N*	*M*(*SD*)	*N*
Gratitude	AI	Deontology	1.53(0.89)	57	3.07(1.42)	71
		Utilitarianism	2.22(1.20)	77	2.35(1.25)	75
	Human driver	Deontology	1.89(1.29)	61	1.89(1.05)	61
		Utilitarianism	2.36(1.27)	59	3.63(1.30)	62
Elevation	AI	Deontology	1.82(1.10)	57	2.30(1.34)	71
		Utilitarianism	2.18(1.23)	77	2.77(1.38)	75
	Human driver	Deontology	2.00(1.22)	61	1.84(1.05)	61
		Utilitarianism	2.37(1.31)	59	3.02(1.36)	62
Pride	AI	Deontology	1.46(0.83)	57	1.77(1.02)	71
		Utilitarianism	1.83(1.08)	77	2.76(1.44)	75
	Human driver	Deontology	1.75(1.22)	61	1.54(0.92)	61
		Utilitarianism	1.93(1.26)	59	2.97(1.35)	62
Sympathy	AI	Deontology	3.58(1.46)	57	3.70(1.13)	71
		Utilitarianism	3.97(1.04)	77	2.45(1.21)	75
	Human driver	Deontology	3.98(1.18)	61	3.48(1.16)	61
		Utilitarianism	4.39(0.85)	59	4.05(1.08)	62
Contempt	AI	Deontology	2.91(1.35)	57	2.13(1.18)	71
		Utilitarianism	1.97(1.14)	77	2.49(1.57)	75
	Human driver	Deontology	2.66(1.35)	61	2.77(1.35)	61
		Utilitarianism	1.75(0.92)	59	1.60(0.91)	62
Anger	AI	Deontology	3.35(1.23)	57	2.49(1.24)	71
		Utilitarianism	2.78(1.26)	77	2.72(1.55)	75
	Human driver	Deontology	3.20(1.30)	61	3.07(1.25)	61
		Utilitarianism	2.27(1.20)	59	2.06(1.21)	62
Disgust	AI	Deontology	3.25(1.20)	57	2.49(1.23)	71
		Utilitarianism	2.43(1.19)	77	2.71(1.48)	75
	Human driver	Deontology	2.98(1.40)	61	2.82(1.25)	61
		Utilitarianism	2.32(1.24)	59	1.74(1.16)	62
Shame	AI	Deontology	2.84(1.18)	57	3.25(1.33)	71
		Utilitarianism	2.78(1.31)	77	1.92(1.21)	75
	Human driver	Deontology	2.62(1.23)	61	3.05(1.31)	61
		Utilitarianism	2.68(1.18)	59	1.69(0.97)	62
Guilt	AI	Deontology	3.91(1.21)	57	3.82(1.23)	71
		Utilitarianism	3.71(1.22)	77	1.88(1.09)	75
	Human driver	Deontology	3.56(1.31)	61	3.64(1.13)	61
		Utilitarianism	3.69(1.32)	59	2.76(1.24)	62
Fear	AI	Deontology	3.98(1.01)	57	3.51(1.30)	71
		Utilitarianism	3.69(1.24)	77	3.71(1.27)	75
	Human driver	Deontology	3.51(1.22)	61	3.41(1.20)	61
		Utilitarianism	3.37(1.46)	59	3.02(1.22)	62

## Appendix E

**Table A4:** The ratings of perceived moral agency in Experiment 1 and Experiment 2.

			Perceived Moral Agency	
Experiment	Decision-Maker	Moral Belief	*M*(*SD*)	*N*
1	AI	Deontology	2.46(1.10)	57
		Utilitarianism	3.43(0.87)	77
	Human driver	Deontology	2.86(1.08)	61
		Utilitarianism	3.90(0.65)	59
2	AI	Deontology	2.80(0.99)	71
		Utilitarianism	3.30(1.06)	75
	Human driver	Deontology	2.68(0.97)	61
		Utilitarianism	4.34(0.47)	62
